# On the Periodic Discharge of Ova, and the Function of Menstruation

**Published:** 1845-08-01

**Authors:** 


*On the periodic discharge of Ova, and the function of Menstruation.*—The following propositions embody the most important conclusions that have been formed by the best authorities on this highly curious subject:

1—Menstruation commences at the period of the *maturity* of the ovules. 2—The final cessation of the catamenial secretion coincides with the abolition of the formative function of the germs. 3—The ovaries of women who have ceased to menstruate, never contain the appearance of any vesicles that have recently burst, or that are about to do so.—4—At each menstrual period, the highly excited state of the ovaries induces in the female a decided propension to coition. 5—The aptitude for fecundation is greatest on those days that immediately preceed the menstrual discharge. 6—In all the lower animals the ovaria becomes tumid during the season of rutting. 7—Women in whom there is a congenital absence of the uterus, but in whom the ovaria are normally developed, experience every month, all the phenomena of menstruation; the sanguineous discharge alone excepted. 10—The catamenial secretion ceases completely in women in whom the ovaria have become affected with organic degeneration. 11—It has been asserted by some writers, that lascivious girls have in this respect like the common hen—occasionally discharged ova from the vagina, and that a mere voluptuous thought will suffice *pour ebranler*, these minute vesicles. 12—In very many women the menstrual period is preceeded by severe colicky pains, attributable most likely, to the turgid and excited state of the ovaries. 13—In those who suffer much at these periods, the cavity of the uterus sometimes becomes lined with a soft flocky membrane — a genuine *membrana caduca*—the formation of which is entirely independant of coition. 14—Lastly in that singular case of monstrosity, in which two girls, Helen and Judith, were united to each other by the posterior and lower part of the back—the catamenial discharge took place in different quantities and at different times from each subject, although there was a complete anastamosis between the abdominal vessels of the two—*memoire pour servir a l'etude des maladies des ovaires, par Achille Chereau.* Paris, 1844.—In Medico-Chirurg—Review for April, 1845.

The general fact that the ova, as it were, ripen and become detached periodically, independently of sexual congress, and that menstruation is a function coincident with, and dependent on this event, seems now to be generally accredited by physiologists. The fact is interesting as serving to confirm the identity between the reproductive functions of the mammiferous and oviparous classes. In addition to its physiological relations, it is both interesting and important in a pathological point of view; and to these we may perhaps add, that it may be found to have certain relations of a practical character, which, in connection with that faculty of the mind termed by Phrenologists philoprogenitiveness, may affect for good or evil, society, and domestic life.

